# Direct Maxillary Sinus Tissue Analysis for *TAS2R38* Polymorphisms: Establishing a Tissue-Based Translational Framework in Odontogenic Rhinosinusitis

**DOI:** 10.3390/jcm15124836

**Published:** 2026-06-22

**Authors:** Andra-Lavinia Greța-Oanță, Alexandra Roman, Ioana Berindan-Neagoe, Ștefan Strilciuc, Ștefan Cristian Vesa, Laura Ancuța Pop, Veronica Elena Trombitaș, Silviu Albu

**Affiliations:** 1 II^nd^ Department of Otorhinolaryngology, Faculty of Dental Medicine, “Iuliu Hațieganu” University of Medicine and Pharmacy, 400349 Cluj-Napoca, Romania; 2 Integrated Outpatient Clinic, Cluj-Napoca Clinical Hospital for Infectious Disease, 400003 Cluj-Napoca, Romania; 3 Department of Periodontology, Faculty of Dental Medicine, “Iuliu Hațieganu” University of Medicine and Pharmacy, 400012 Cluj-Napoca, Romania; 4 Genomics Department, MEDFUTURE Institute for Biomedical Research, “Iuliu Hațieganu” University of Medicine and Pharmacy, 400132 Cluj-Napoca, Romania; 5 Department of Pharmacology, Toxicology and Clinical Pharmacology, “Iuliu Hațieganu” University of Medicine and Pharmacy, 400337 Cluj-Napoca, Romania

**Keywords:** odontogenic rhinosinusitis, bitter taste receptor, *TAS2R38*, innate immunity, chronic rhinosinusitis

## Abstract

**Background/Objectives:** Bitter taste receptors (T2Rs), specifically T2R38, are present in the respiratory epithelium and react with bacterial quorum-sensing molecules to induce an innate immunity response. Although *TAS2R38* polymorphisms have been correlated with susceptibility to chronic rhinosinusitis (CRS), they have not yet been explored in odontogenic rhinosinusitis (ORS), a distinct form of CRS with particular microbial and inflammatory features. We aim to establish a proof-of-concept methodology for investigating *TAS2R38* genetic variants in ORS using direct maxillary sinus tissue analysis and demonstrate the feasibility of this translational approach. **Methods:** We conducted a prospective pilot case–control study of 36 ORS patients and 37 controls undergoing septoplasty without sinonasal disease. Maxillary sinus mucosal biopsies were obtained intraoperatively with informed consent. Genomic DNA was extracted using the PureLink Genomic DNA Mini Kit and quantified via NanoDrop spectrophotometry. *TAS2R38* haplotypes were determined and classified as taster (PAV/PAV), non-taster (AVI/AVI), or intermediate (PAV/AVI) phenotype. **Results:** Among fully classifiable canonical TAS2R38 phenotypes (32 ORS patients, 28 controls), distributions were: tasters 12.5% vs. 25.0%, non-tasters 31.3% vs. 25.0%, and intermediate 56.3% vs. 50.0%. AVI/AVI non-taster status was not significantly associated with ORS susceptibility (OR = 1.36, 95% CI: 0.44–4.25; Fisher’s exact *p* = 0.775). **Conclusions:** This proof-of-concept study demonstrates that genotyping-grade genomic DNA can be recovered from acutely inflamed maxillary sinus mucosa, validating this substrate for future tissue-based expression, functional, and microbiome analyses not obtainable from peripheral samples; germline genotyping itself does not require sinus tissue. The observed difference in non-taster prevalence (31.3% vs. 25.0%) did not reach statistical significance and is reported descriptively. This directional trend is hypothesis-generating only and, given the limited statistical power, does not constitute evidence for an association. The demonstrated feasibility, together with the established biological rationale, supports an adequately powered confirmatory study and lays the foundation for future investigation of taste receptor genetics in ORS pathogenesis, and potentially personalized therapeutic strategies.

## 1. Introduction

Chronic rhinosinusitis (CRS) is a frequent inflammatory condition of the nasal and paranasal sinus mucosa, affecting approximately 11% of the adult population and significantly impacting quality of life and healthcare systems worldwide [1].

ORS represents a distinct clinical subtype among CRS, accounting for 25% to 40% of all chronic maxillary sinusitis [2,3], most commonly occurs unilaterally [4,5,6], and represents 45% to 75% of unilateral maxillary sinus opacification on computed tomography (CT).

Etiology is attributed to dental infections, iatrogenic injury during dental procedures, or the migration of dental materials into the maxillary sinus, periodontal disease that had perforated the Schneiderian membrane, implant procedures including the increasingly frequent maxillary grafting, irritation and secondary infection caused by intra-antral foreign bodies, with no prior sinonasal inflammation [7,8,9].

The prevalence of this pathology is frequently underestimated, according to Wuokko-Landén et al., and approximately 15% of acute rhinosinusitis may be odontogenic [10]. Regarding CRS, approximately a quarter of the diagnosed cases could be of dental causes [11].

ORS typically involves polymicrobial aerobic–anaerobic bacteria of oral origin, with the most frequently found being *Peptostreptococcus* spp, Prevotella intermedia, and Fusobacterium [11], and presents unique microbiological and radiological features, distinguishing it from classical CRS [2,9,12]. The canonical activators of T2R38 are acyl-homoserine lactones (AHLs), principally 3-oxo-C12-HSL, and quinolones produced by Gram-negative aerobes such as *Pseudomonas aeruginosa*. The dominant ORS pathogens, Fusobacterium nucleatum, Prevotella intermedia, and *Porphyromonas gingivalis*, are obligate anaerobes that rely instead on the LuxS/AI-2 quorum-sensing system, generating S-THMF-borate as their principal signaling molecule. Whether AI-2 or related anaerobic metabolites can functionally activate T2R38 or homologous receptors has not been established. Short-chain fatty acids (SCFAs), particularly butyrate and propionate, abundant fermentation end-products of all three genera, represent a plausible but lower-potency alternative activation route. This mechanistic gap between the ORS microbiome and the known T2R38 agonist repertoire represents a critical unresolved question that the present study explicitly acknowledges and that future functional investigations must address.

Management of ORS involves a shared decision-making process between the otolaryngologist and the dentist [3]. However, interdisciplinary collaboration is not always easy to achieve, and ORS can become difficult to treat.

In recent years, bitter taste receptors (T2Rs), particularly T2R38, have emerged as key components in the innate immunity of the upper airways and gastrointestinal system. These receptors, initially described in gustatory cells [13], are also expressed in the respiratory epithelium where they detect bacterial quorum-sensing molecules and trigger nitric oxide production, mucociliary clearance, and antimicrobial responses [4,5,6,12,13]. Based on existing studies, the presence of bitter taste receptors may represent a viable therapeutic target in the treatment of CRS. Several studies have demonstrated that certain *TAS2R38* genotypes, especially the non-functional AVI/AVI variant, are linked to increased susceptibility to CRS and complications after sinus surgery [14,15,16,17]. However, the role of bitter taste receptors in odontogenic rhinosinusitis has not been explored to date.

Bitter taste receptors (T2Rs) are G protein-coupled receptors (GPCRs) found in ciliated epithelial cells and solitary chemosensory cells (SCCs) of the upper respiratory tract. T2R38, one of the most studied bitter taste receptors, can recognize acyl-homoserine lactones (AHLs) and quinolones, signaling molecules secreted by Gram-negative bacteria such as *Pseudomonas aeruginosa* [18]. Within the precision medicine framework, the taster, non-taster, and intermediate classifications represent innate functional phenotypes rather than clinical phenotypes, as they define mechanistically distinct subtypes of host defense capacity rather than observable clinical presentations.

**Figure 1:**
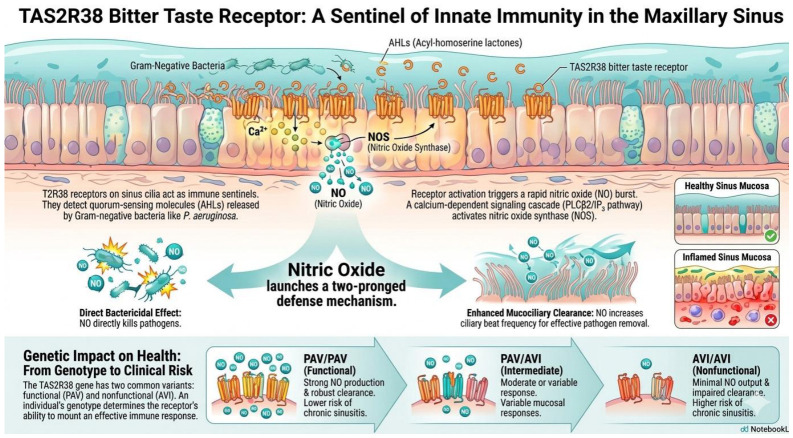
The implications of *TAS2R38* bitter taste receptors in the immunity of the maxillary sinus.

Given the distinct etiopathogenesis of ORS, which involves direct exposure of the sinus mucosa to oral pathogens and dental infection and inflammation, we hypothesized that genetic variations in bitter taste receptors may influence individual susceptibility to ORS. This pilot, proof-of-concept study has two primary objectives: to establish the methodological feasibility of recovering and genotyping *TAS2R38* from acutely inflamed maxillary sinus mucosa in an ORS population; and to generate preliminary non-taster prevalence estimates to inform power calculations for a future confirmatory trial. Hardy–Weinberg equilibrium was verified in both groups as a routine quality control step (see Statistical Analysis). Comparison of *TAS2R38* genotype distributions between ORS patients and controls with septal deviation serves as an exploratory secondary endpoint, with the study explicitly underpowered for definitive epidemiological inference. We emphasize that germline *TAS2R38* genotyping does not require sinus tissue and is obtainable from blood, saliva, or buccal swab. The rationale for using maxillary sinus mucosa is to establish that genotyping-grade genomic DNA can be recovered from acutely inflamed, protease-rich sinus tissue under routine surgical conditions—the necessary first step toward an integrated genotype–expression–function–microbiome platform that requires in situ tissue and cannot be reproduced from peripheral samples (Figure 1).

## 2. Materials and Methods

### 2.1. Study Design

We performed a prospective study from February 2023 to November 2024 where we enrolled subjects presenting with odontogenic sinusitis. Eligible patients presenting during the study period were consecutively enrolled to ensure an unbiased and representative sample.

Inclusion criteria—Odontogenic maxillary sinusitis group

- Men and women aged 18–65 years who provided explicit consent for harvesting mucosal biopsy from the affected sinus during surgery for histologic or molecular analysis.
- Diagnosis of odontogenic maxillary sinusitis confirmed by CT scan, reevaluated at admission by the attending surgeon.
- Lund–Mackay score > 3 on the affected sinus.
- Complete endoscopic evaluation confirming sinus involvement.
- Indication for Functional Endoscopic Sinus Surgery (FESS).
- Presence of at least one symptom for ≥6 weeks including: ongoing purulent rhinorrhea, facial pressure, foul odor, nasal obstruction.
- History of previous sinus surgery permitted.
- Non-smoker.

Exclusion criteria—Odontogenic maxillary sinusitis group

- Age <18 or >65.
- Active or chronic immune disorders or current immunosuppressive therapy.
- Acute upper respiratory infection at the time of enrollment.
- Smokers.
- Pregnancy or lactation.

Control group—Inclusion criteria

- Men and women aged between 18 and 65 undergoing septoplasty secondary to septal deviation.
- Absence of clinical or endoscopic signs of sinus disease at the time of evaluation.
- Willingness to provide informed consent for use of biopsy material in research.
- No requirement for CT imaging beyond clinical indications, in accordance with ethical standards.
- Not age- or sex-matched to the study group but recruited from the same patient population.
- Septal mucosal biopsy obtained from standardized anterior septal location to ensure sampling consistency.

Control group—Exclusion criteria

- Patient with history of endoscopic evidence of acute or chronic rhinosinusitis.
- Previous sinonasal or maxillofacial surgery.
- Systemic inflammation or immunologic disorders.
- Recent prescribed or self-administered antibiotic or corticosteroid therapy—4 weeks prior to surgery.
- Smokers.
- Pregnancy or lactation.

### 2.2. Ethical Approval and Consent

All procedures involving human participants were conducted in agreement with the ethical standards of the Institutional Research Committee of The University of Medicine and Pharmacy “Iuliu Hațieganu” (Approval No. AVZ12/2023-01-11) and with the 1964 Helsinki declaration and its later amendments. Written informed consent was obtained from all participants prior to enrollment. Biopsies were performed under general anesthesia, minimizing discomfort and bleeding. Participants provided specific consent for genetic testing and storage of genomic material. No financial compensation was offered; participation was voluntary.

#### 2.2.1. Sample Collection and Preservation

The biologic material was preserved as it was harvested, within 5 min of excision, in a liquid nitrogen container, transported, stored and processed together at the Genomics Department, MEDFUTURE Institute for Biomedical Research at the University of Medicine and Pharmacy “Iuliu Hațieganu”, Cluj Napoca. Sterile and surgical instruments and DNase/RNase-free 2 mL tubes were used. Each sample was labeled with a unique identifier to maintain confidentiality. Samples were stored at −196 °C and processed in one batch within 24 months of collection.

#### 2.2.2. DNA Extraction

Up to 25 mg of tissue was used per extraction. DNA was extracted from tissue samples from 36 cases and 37 controls using the PureLink Genomic DNA Mini Kit (ThermoFisher Scientific, Waltham, MA, USA). The extracted DNA was quantified using the NanoDrop spectrophotometer (ThermoFisher Scientific, Waltham, MA, USA) and the DNA concentration was between 6.2 and 435.9 ng/µL.

#### 2.2.3. SNP Genotyping Assay

For the genotyping analysis, we analyzed the *TAS2R38* rs713598, rs1726866, and rs10246939 using three different SNP assays: c_8876467_10, c_9506827_10, and c_9506826_10 from ThermoFisher Scientific (Waltham, MA, USA). These SNPs were selected due to their known functional relevance in bitter taste receptor signaling and potential modulation of sinonasal immunity. Mainly, we used 40 ng/µL of DNA, 5 µL of TaqMan Genotyping Master mix 2X (ThermoFisher Scientific, Waltham, MA, USA), 0.5 µL of SNP assay 20X, and 3.5 µL of DNase-free water. The reactions were amplified on the ViiA 7 real-time PCR instrument using the following protocol: 1 cycle—60 °C–30 s; 1 cycle—95 °C–10 min, 45 cycles—95 °C–15 s and 60 °C–1 min, 1 cycle—60 °C–30 s. The data analysis was done on the instrument’s software, which discriminates between homozygote for the wildtype allele, and heterozygote or homozygote for the mutant allele.

### 2.3. Quality Control and Blinding

Laboratory personnel performing the DNA extraction and genotyping were aware of the case or control status of the samples. They were blinded to the expected genotypes and the study hypotheses, reducing the risk of observer bias. All samples were processed simultaneously using the same kit and reagents, ensuring uniform handling and minimizing batch effects. This approach maintained procedural consistency across all samples and strengthened the reliability and comparability of genotyping results between cases and controls.

## 3. Results

In the odontogenic sinusitis group, we found four (11.1%) taster (PAV/PAV), 10 (27.8%) non-taster (AVI/AVI), 18 (50%) intermediate (PAV/AVI), two (5.6%) mixed, and two (5.6%) incomplete. For the control group, there were seven (18.9%) tasters, seven (18.9%) non-tasters, 14 (37.8%) intermediate, six (16.2%) mixed, and three (8.1%) incomplete.

Mixed genotypes were those that did not conform to the canonical PAV or AVI haplotype patterns across the three SNPs—for example, atypical or inconsistent SNP combinations, possible recombination variants, genotyping errors, or rare haplotypes not covered by the PAV/AVI nomenclature. Incomplete genotypes were those with missing data for one or more SNPs.

Because mixed and incomplete genotypes cannot be equated with canonical intermediate (PAV/AVI) haplotypes, they were reported separately and excluded from the primary phenotype-based comparison; they were retained within the intermediate category only in a sensitivity analysis (see Statistical Analysis). After their exclusion, the primary comparison comprised 32 ORS patients and 28 controls with fully classifiable canonical *TAS2R38* phenotypes.

Genotype distributions did not deviate significantly from Hardy–Weinberg equilibrium in either group.

### Statistical Analysis

All statistical analyses were conducted using MedCalc^®^ Statistical Software version 23.3.7 (MedCalc Software Ltd., Ostend, Belgium; https://www.medcalc.org; 2025). SNP genotype frequencies in cases and controls were tested for Hardy–Weinberg equilibrium as a pre-analysis quality control step. For the between-group comparison, *TAS2R38* phenotypes were dichotomized as non-taster (AVI/AVI) versus all other phenotypes (taster [PAV/PAV] and intermediate [PAV/AVI] combined), and the two groups were compared using Fisher’s exact test. Genotypes that could not be assigned to a canonical PAV/AVI haplotype (“mixed”) or had incomplete SNP data (“incomplete”) were excluded from this primary comparison; a sensitivity analysis retaining them within the intermediate category was performed to assess robustness. A *p* value < 0.05 was considered statistically significant.

**Figure 2:**
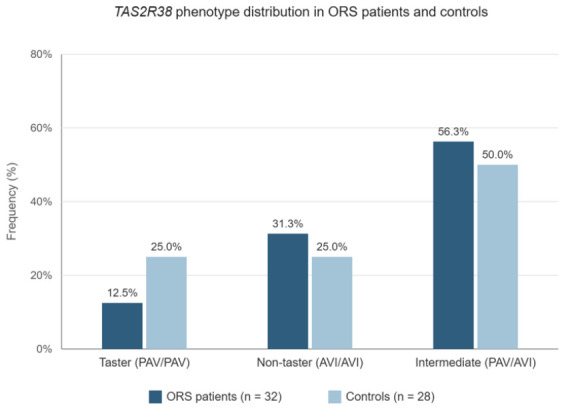
Proportions of canonical *TAS2R38* taste phenotypes among ORS patients (n = 32) and controls (n = 28). Mixed and incomplete genotypes were excluded from the figure and from the primary phenotype-based comparison. AVI/AVI non-taster status was more frequent in ORS patients than in controls, but the difference was not statistically significant (31.3% vs. 25.0%; OR = 1.36, 95% CI: 0.44–4.25; Fisher’s exact *p* = 0.775).

In the primary comparison of fully classifiable canonical phenotypes (32 ORS patients, 28 controls), non-taster (AVI/AVI) status was not significantly associated with ORS susceptibility (OR = 1.36, 95% CI: 0.44–4.25; Fisher’s exact *p* = 0.775), reflecting a non-significant trend toward higher non-taster prevalence in the ORS group. The sensitivity analysis retaining mixed and incomplete genotypes within the intermediate category (36 ORS patients, 37 controls) gave a concordant, non-significant result (OR = 1.4, 95% CI: 0.5–3.9; Fisher’s exact *p* = 0.595) (Figure 2).

To inform the design of a future confirmatory multicenter trial, prospective sample size estimates were calculated across a range of clinically plausible effect sizes (Table 1). Calculations were based on a two-sided Fisher’s exact test at α = 0.05, using a baseline non-taster prevalence of 25.0%, as observed among controls with fully classifiable canonical phenotypes in the primary analysis. The primary planning assumption was a target OR of 2.0, consistent with the effect sizes reported in the *TAS2R38*–CRS literature; the observed OR of 1.36 from this pilot is presented for reference only and was not used as the basis for sample size estimation, given the well-recognized limitations of post hoc power calculations in underpowered exploratory studies. Because the primary analysis excludes genotypes that cannot be assigned a canonical phenotype, a conservative 20% attrition correction was applied to all estimates, reflecting the combined rate of unclassifiable (mixed and incomplete) genotypes observed in the current cohort (17.8% overall; 11.1% in ORS patients and 24.3% in controls). At 80% power, approximately 165 analyzable participants per group are required; after the 20% attrition correction, the corresponding recruitment target is approximately 207 per group (414 total). At 90% power, approximately 216 analyzable participants per group are required, corresponding to a recruitment target of approximately 270 per group (540 total).

## 4. Discussion

The role of polymicrobial biofilms has been a major focus of discussion in the scientific literature, particularly concerning ORS cases that show resistance to treatment. Bacterial biofilms are dynamic, multispecies microbial communities in which bacteria replicate, maintain metabolic activity, and become embedded in a matrix composed primarily of exopolysaccharides, proteins, and nucleic acids [5].

Troeltzsch et al. conclude that the pathologies associated with dental implants are recognized as important etiological factors in maxillary sinusitis that necessitate surgical management [7]. Until recently, odontogenic rhinosinusitis was classified within the broader framework of chronic rhinosinusitis and largely regarded as an anatomical variant defined by its dental origin rather than as a pathophysiologically distinct condition [1]. This classification has increasingly been questioned as accumulating clinical, microbiological, and immunological evidence suggests that ORS is not a uniform entity, but rather an umbrella term encompassing several mechanistically distinct subtypes determined by their underlying dental cause. The main categories that are periapical pathology, periodontal disease, implant-related sinusitis, and iatrogenic post-surgical disease, differ substantially in terms of the microbial inoculum introduced into the sinus, the chronicity of bacterial translocation, and the tissue environment in which the inflammatory response develops.

Periapical pathology typically introduces a predominantly polymicrobial anaerobic immunostimulant through direct apical-to-sinus fistulization, most often resulting in a chronic, low-grade inflammatory profile [19]. Periodontal disease, in contrast, introduces a biofilm community that is already shaped by subgingival dysbiosis, commonly enriched in *Porphyromonas gingivalis*, *Treponema denticola*, and *Tannerella forsythia*, and characterized by the well-documented immune-modulating and immune-evasive properties of this microbial conglomerate [20]. Implant-related sinusitis, an increasingly recognized subtype in the era of reconstructive dentistry [7], represents a more complex scenario in which infectious processes coexist with mechanical irritation and foreign body-driven immune activation, including increased expression of IL-4 and IL-13, creating an inflammatory environment that is not fully captured by existing CRS endotype frameworks [21]. Iatrogenic ORS, typically arising from sinus floor perforation or sinus-lifting procedures, represents a temporally defined acute inoculation event occurring in a previously healthy sinus and may therefore be more easily resolved once the dental source has been eliminated than more chronic subtypes.

These etiological differences have important implications for the inflammatory profile of the sinus mucosa and, importantly, for research on the *TAS2R38* genotype. The capacity of the colonizing microbial community to activate T2R38 signaling is likely to vary across these etiological subtypes, meaning that the functional impact of PAV/PAV versus AVI/AVI genotype status may not be uniform throughout ORS. For this reason, future studies should incorporate structured etiological stratification as a central analytical variable rather than treating ORS as a single, homogeneous condition. Such an approach may help clarify genotype–phenotype relationships that could otherwise remain obscured in aggregated analyses.

Odontogenic maxillary sinusitis is known to be enriched for anaerobes such as *Fusobacterium*, *Prevotella*, *Porphyromonas* and mixed anaerobic flora, with lower prevalence of *Staphylococcus* and *Pseudomonas* compared with CRS [22,23,24]. In CRS, the predominant bacteria found are *Staphylococcus* aureus and anaerobes (*Prevotella*, *Porphyromonas*, *Fusobacterium*, *Peptostreptococcus*), with Gram-negative rods appearing more frequently in nosocomial or high-risk settings [25,26,27]. Recent meta-analyses and microbiome studies confirm the distinct profile, with Fusobacterium and anaerobes being indicators for odontogenic disease [23,26].

Human neutrophils express functional T2R38 receptors. The *Pseudomonas aeruginosa* quorum-sensing molecule 3-oxo-C12-HSL (AHL-12) is internalized and co-localizes with T2R38, with direct interaction confirmed by pull-down assays. Blocking T2R38 reduces AHL-12 binding and activation, indicating that T2R38 functions as a receptor for this AHL molecule [28,29]. Structural and functional studies of airway bitter taste receptors have shown that 3-oxo-C12-HSL and C8-AHL can activate other receptors in this family, including T2R4, T2R14, and T2R20, at a micromolar higher potency. These interactions involve defined binding sites located in extracellular loop 2 of the receptors [30]. Available evidence indicates that T2R38, together with other T2Rs, contributes to the detection of AHL-mediated signaling from Gram-negative bacteria including *Pseudomonas aeruginosa* [28,29,30,31]. T2R38 activation differs qualitatively by bacterial group: Gram-negative pathogens primarily engage T2R38 via specific quorum-sensing signals (AHLs, quinolones), whereas Gram-positive-rich communities appear to activate T2R38 via broader small-molecule metabolites rather than known peptide QSMs [32,33].

A key mechanistic consideration arising from the microbial profile of ORS concerns the capacity of the dominant odontogenic anaerobes to engage T2R38-mediated innate defenses. The existing literature on T2R38 activation is based on *Pseudomonas aeruginosa*, where the 3-oxo-C12-HSL serves as the canonical agonist. In contrast, the main organisms in ORS, *Fusobacterium nucleatum*, *Prevotella intermedia*, and *Porphyromonas gingivalis*, are Gram-negative anaerobes that do not utilize classical AHL-based quorum-sensing. Instead, they primarily use the LuxS/AI-2 system, generating (2S,4S)-2-methyl-2,3,3,4-tetrahydroxytetrahydrofuran borate (S-THMF-borate) as their principal interspecies signaling molecule [34,35], along with species-specific effectors such as gingipain proteases and d-amino acid-containing peptides from *P. gingivalis*. Whether these non-AHL quorum-sensing molecules can functionally activate T2R38 or related receptors (T2R4, T2R14, T2R20) remains largely unexplored.

Interestingly, short-chain fatty acids, particularly butyrate and propionate, which are abundant end-products of anaerobic fermentation by all three genera, have been shown to activate bitter taste receptors at physiologically relevant concentrations [36]. This suggests a potential alternative pathway for T2R engagement in the anaerobe-rich ORS microenvironment. Additionally, *P. gingivalis* can subvert pattern recognition pathways through its atypical lipid A structure, acting as a TLR4 antagonist rather than an agonist [37,38], while its gingipain proteases actively degrade complement components and immunoglobulins, further attenuating mucosal defense [20].

In polymicrobial ORS biofilms, cross-species AI-2 signals can modulate gene expression across species, and minor aerobic or facultatively anaerobic Gram-negative constituents, such as *Pseudomonas* or *Haemophilus,* which may generate AHL signals sufficient to engage T2R38, even if they are not numerically dominant. Together, these observations suggest that T2R38 may function less as a direct sentinel of the primary ORS pathogens and more as a detector of the broader quorum-sensing environment within the polymicrobial biofilm. In this framework, the protective advantage of the PAV/PAV functional genotype in the maxillary sinus would operate through enhanced NO-driven mucociliary clearance in response to metabolic cues from the biofilm, rather than through direct recognition of species-specific AHLs. Conversely, AVI/AVI non-tasters, lacking this signal amplification, may be more susceptible to persistent colonization by anaerobe-dominated communities, because these organisms are relatively weak activators of T2R38 through canonical pathways. This creates a selective susceptibility that is mechanistically distinct from but conceptually parallel to the Gram-negative vulnerability observed in CRS. This hypothesis is biologically testable and provides a strong rationale for integrating sinus microbiome sequencing with T2R functional phenotyping in future ORS cohort studies.

Current evidence supports immunological distinctions between odontogenic rhinosinusitis and classic chronic rhinosinusitis. Histopathological studies show that eosinophilic infiltration, although present in a substantial proportion of ORS cases (39.1%), occurs significantly less frequently than in chronic rhinosinusitis with nasal polyps (CRSwNP), where it is reported in 63.2% of cases (*p* < 0.05). In addition, ORS demonstrates lower levels of squamous metaplasia and fibrosis compared with CRSwNP, and its inflammatory pattern more closely resembles that observed in chronic rhinosinusitis without nasal polyps (CRSsNP), often accompanied by pronounced acute-on-chronic inflammatory changes [39]. By contrast, CRSwNP is typically associated with type 2 inflammation, characterized by eosinophil-dominant tissue infiltration, submucosal edema, and increased expression of IL-4, IL-5, and IL-13 [40,41,42]. This immunological profile is not consistently observed in ORS.

Mucociliary dysfunction is a well-established contributor to chronic rhinosinusitis pathogenesis, but its role in ORS may be comparatively less pronounced. Histological studies have suggested that the Schneiderian membrane in ORS may exhibit relatively preserved epithelial barrier integrity, reflected by increased expression of tight junction proteins [40]. However, direct functional comparisons of mucociliary clearance between odontogenic and non-odontogenic types of sinusitis remain limited in the current literature, highlighting an important methodological gap.

Taken together, the microbiological, immunological, and genetic evidence supports a coherent model of ORS that is mechanistically distinct from classic CRS, justifying its conceptualization as an independent disease entity rather than a mere anatomical variant of chronic rhinosinusitis. The initiating event, odontogenic bacterial translocation into the maxillary sinus, introduces a polymicrobial, anaerobe-dominated community [22,23,24] that, as described above, relies on LuxS/AI-2 and SCFA-mediated intercellular signaling rather than AHL chemistry. This has a direct consequence for first-line epithelial defense: the NO-driven mucociliary clearance axis [43,44] receives a comparatively weak activating stimulus from the dominant ORS flora, even in PAV/PAV individuals, as SCFA-mediated T2R engagement provides only a lower-potency compensatory signal insufficient to substitute for AHL-driven activation. In AVI/AVI non-tasters, this already attenuated response is further diminished by intrinsically reduced T2R38 receptor function [16,45], producing a compounded innate defense deficit that is qualitatively and quantitatively distinct from that observed in Gram-negative-dominant CRS. The downstream immunological consequence of this failure of early innate clearance is not type 2 polarization—the absence of sufficient aeroallergen or staphylococcal superantigen exposure precludes the IL-4/IL-5/IL-13 axis characteristic of CRSwNP [40,41,42]—but rather the neutrophil-dominant, acute-on-chronic inflammatory state histopathologically established above [39,40]. We propose that the preserved epithelial tight junction integrity observed in ORS should not be interpreted as a marker of immunological competence. Rather, it reflects a fundamentally different failure mode: not barrier destruction driven by eosinophil-mediated remodeling, but clearance paralysis resulting from insufficient T2R38 engagement during the critical early window of colonization, a deficit further amplified by the active immune subversion mechanisms of *P. gingivalis* described above [20,37,46]. We further propose that the *TAS2R38* genotype operates not as a binary susceptibility switch but as a quantitative modulator of the innate response threshold, with AVI/AVI individuals positioned at the vulnerable end of a continuous spectrum shaped by both receptor function and the intrinsically low T2R38-activating potential of the colonizing microbiota. Clinically, this model predicts that AVI/AVI patients with odontogenic implant-related or periodontal sinusitis, where the anaerobe burden is highest, represent the subgroup at greatest risk of biofilm persistence, medical treatment failure, and disease recurrence following surgical intervention [7,16,45], and therefore the population most likely to benefit from adjunctive T2R agonist-based topical therapy aimed at pharmacologically restoring receptor-mediated innate signaling. Within the broader classification of allergic sinusitis proposed by Olivier, ORS associated with the AVI/AVI *TAS2R38* polymorphism may represent a non-IgE-mediated innate hypersensitivity endotype, mechanistically distinct from both classical IgE-mediated allergic rhinosinusitis and the type 2 eosinophilic endotype of CRSwNP [47].

Starting with the established evidence that *TAS2R38* polymorphisms influence the course of chronic rhinosinusitis and upper airway defensive mechanisms [48,49], the present study establishes the first methodological framework for investigating bitter taste receptor T2R38 and related T2Rs on cilia from maxillary, ethmoid, sphenoid, and middle turbinate epithelium, confirming that the NO mucociliary clearance axis operates directly within the maxillary sinus mucosa [43]. Given the relatively dependent drainage anatomy of the maxillary sinus and its high predilection for stagnant mucus and biofilm accumulation, a pathway coupling bacterial sensing to rapid NO release and ciliary acceleration is particularly critical for local clearance and biofilm control [44,50]. Functional T2R38 (PAV/PAV) is associated with better bacterial clearance, fewer infections, and reduced need for sinus surgery, whereas AVI/AVI genotypes are linked to more frequent Gram-negative infection, biofilm formation, and medically recalcitrant CRS [16,18,44,45]. These associations are derived largely from maxillary and other sinus tissue in surgical CRS cohorts and have not been established in ORS.

*TAS2R38* genotype-based risk stratification holds potential for predicting susceptibility to maxillary sinus infection and likelihood of surgical intervention [16]. T2R agonists—including quinine and flavones—increase NO production and ciliary beating in sinonasal cultures and are under investigation as topical therapies to enhance innate maxillary sinus defenses, particularly in patients with attenuated T2R38 function [46].

This study characterizes the *TAS2R38* genotype without a corresponding functional phenotypic assessment, representing an important methodological boundary that warrants explicit acknowledgment. The relationship between germline genotype and receptor functional output is not strictly deterministic: post-translational mechanisms including glycosylation, membrane trafficking efficiency, and G protein-coupling fidelity can modulate T2R38 activity independently of haplotype status. In the acutely inflamed ORS sinus environment, characterized by elevated IL-1β, TNF-α, and IL-17A, receptor expression may be further downregulated at the transcriptional level. Thus, even PAV/PAV individuals could exhibit attenuated T2R38 signaling during acute-on-chronic inflammatory episodes. On the opposite spectrum, post-surgical resolution may restore receptor expression closer to genotype-predicted levels, such that functional phenotyping performed exclusively at the time of surgery may not accurately reflect a patient’s baseline immunological capacity. Validated strategies exist to bridge this genotype–phenotype gap. Systemic PROP or PTC taste testing provides a rapid, non-invasive surrogate of T2R38 functional status, measuring the observable bitter taste perception response directly and enabling functional stratification of taster versus non-taster phenotype independently of germline genotype, a distinction particularly relevant in the inflamed ORS sinus environment where transcriptional downregulation may decouple receptor expression from haplotype-predicted activity. For sinonasal-specific functional assessment, ex vivo measurement of ciliary beat frequency (CBF) or nasal nitric oxide (nNO) from surgical brushings cultured at the air–liquid interface allows controlled bitter agonist challenge with direct receptor activity quantification, a protocol already established in cystic fibrosis and primary ciliary dyskinesia research programs. Immunofluorescence quantification of T2R38 protein in surgical mucosal specimens, architecturally accessible within the present study’s tissue collection protocol, can provide an intermediate layer of evidence between genotype and functional output, distinguishing whether reduced activity reflects receptor absence, mislocalization, or uncoupling.

Prior genetic studies of *TAS2R38* in chronic rhinosinusitis have relied predominantly on tissue from nasal polyps, inferior turbinate, or ethmoid specimens obtained in the context of non-odontogenic disease. None of these sources reflect the specific pathological microenvironment of the odontogenic maxillary sinus during an active episode, a niche characterized by direct polymicrobial inoculation of oral origin, anaerobe-dominated biofilm formation, and a distinct acute-on-chronic inflammatory milieu. By sampling directly from the diseased sinus mucosa at the time of surgical intervention, this study captures tissue that is contemporaneously exposed to the odontogenic pathogenic stimulus, providing a site-specific biological context that peripheral blood, saliva, or non-odontogenic sinonasal specimens cannot replicate.

The successful extraction of high-quality genomic DNA and reliable *TAS2R38* genotyping from acutely inflamed mucosal tissue, a technically demanding substrate due to elevated protease activity, leukocyte infiltration, and potential DNA degradation, validates the procedural feasibility of recovering genotyping-grade DNA from this difficult substrate, which is the rate-limiting requirement for the downstream tissue-based assays, not for genotyping, which does not require tissue. This represents a meaningful methodological advance, establishing a protocol that can be adopted by future multicenter cohort studies without requiring modifications to standard intraoperative biopsy practice.

Critically, the framework developed here is modular by design. Future iterations of this protocol can incorporate, from the same surgical specimen, immunofluorescence quantification of T2R38 protein expression and subcellular localization; ex vivo ciliary beat frequency measurement and nasal nitric oxide assessment from air–liquid interface cultures of surgical brushings; and concurrent 16S rRNA sinus microbiome profiling. This integration would enable the construction of a genotype–expression–function–microbiome axis within the target organ, a translational platform that is structurally impossible with blood- or saliva-derived genotyping approaches and that addresses the principal mechanistic gaps identified in current ORS research.

The inclusion of only non-smoking patients without autoimmune diseases minimized potential confounders that may alter TAS2R expression, though this approach limited recruitment. This methodological rigor, while difficult for subject enrollment, enhances the translational validity of our protocol despite the limited sample size. Successful DNA extraction and genotyping from inflamed sinus mucosa demonstrates the practicability of this direct tissue approach for future genetic investigations in ORS.

Future research should evolve in several important areas to advance this translational methodology toward clinically meaningful applications. At minimum, a two-tier phenotyping strategy, combining systemic taste testing at enrollment with ex vivo ciliary beat frequency (CBF) or nNO functional assessment from surgical tissue, should be incorporated, enabling the construction of the genotype–expression–function axis currently absent from ORS research. Firstly, enlarging the cohort size is essential to achieve adequate statistical power and adding stratification by established ORS severity classifications and microbiological profiles. Secondly, integrating sinus microbiome sequencing with TAS2R profiling could identify genotype–microbiota interactions influencing disease persistence or recurrence, and would allow prospective validation of the pathophysiological model proposed here. Functional mucociliary assessment across ORS and non-ORS sinusitis subtypes, combined with longitudinal immune endotype profiling following elimination of the odontogenic source, would directly address the methodological gaps identified above. Thirdly, including patients with smoking history or autoimmune diseases in separate analytical cohorts would clarify the impact of these factors upon the genetic patterns. Finally, given that recent single-step protocols combining FESS with concurrent dentoalveolar source control now resolve the large majority of ORS cases [51], longitudinal follow-up of these surgically treated cohorts could clarify whether the *TAS2R38* functional phenotype and genotype predict treatment outcomes, symptom resolution, or disease recurrence in the subset that proves refractory. These research directions provide a clear pathway from methodological validation toward statistically robust and clinically meaningful conclusions, contributing to the broader precision medicine agenda of endotype-specific diagnosis and treatment in inflammatory airway disease advocated by Agache and Akdis [52].

The present study was prospectively designed as a pilot, proof-of-concept investigation, with the primary objectives of: 1. establishing the feasibility of *TAS2R38* genotyping in an odontogenic sinusitis population and 2. generating preliminary non-taster prevalence estimates, to inform future sample size planning. Both objectives were achieved, and HWE was confirmed as a routine quality control step. Therefore, the lack of statistical significance in an underpowered exploratory comparison is uninformative with respect to the underlying biological hypothesis in either direction.

Sample size estimation for a future confirmatory study was based on a pre-specified minimum clinically relevant odds ratio (OR) of 2.0. This threshold was informed by, though not derived from, the existing *TAS2R38*–CRS literature, which demonstrates significant but generally modest genotype associations. Adappa et al. [16] reported a significant enrichment of the AVI/AVI genotype among patients with medically recalcitrant CRS requiring surgery compared with a general population cohort (χ^2^(2) = 6.526, *p* = 0.0383). Subsequent studies by Jeruzal-Świątecka et al. [53] and Dżaman et al. [54] further examined the relationship between *TAS2R38* polymorphisms and sinonasal inflammatory disease; in particular, Jeruzal-Świątecka et al. reported an odds ratio of 1.43 for AVI haplotype frequency increase in CRSwNP compared to controls, indicating a modest but significant increase in risk. Because these reported effects, and our own pilot estimate (OR 1.36, 95% CI 0.44–4.25), cluster below the pre-specified threshold, powering for an OR of 2.0 reflects a pragmatic feasibility compromise; Table 1 additionally reports the substantially larger samples required should the true effect approximate 1.36. The baseline non-taster prevalence used for these estimates (25.0%, observed among classifiable controls) is consistent with the lower bound of the reported European AVI/AVI frequency range of 25–30% [55,56], supporting the representativeness of the control sample and the soundness of the prevalence assumption underlying the calculation.

Our study had several limitations. The relatively low sample size limited the power of the analysis, which means that no firm conclusions about the relation between *TAS2R38* mutations and susceptibility to ORS can be made. The current study should be viewed as a pilot exploratory study. The lack of formal matching of the control and ORS groups according to age and sex criteria is another limitation. However, it is worth noting that such matching would be particularly problematic since rather strict inclusion/exclusion criteria were used in order to exclude any potential biological confounding factors. Another limitation of this study is the exclusive focus on genotyping without taking into account the functional testing of T2R38 receptors. Specifically, neither systemic taste sensitivity testing using 6-n-Propylthiouracil (PROP) or Phenylthiocarbamide (PTC), nor direct sinonasal functional measurements including nasal nitric oxide levels and ciliary beat frequency, were performed. These modalities would be necessary to establish the relationship between genotype and actual receptor functional output, and are incorporated as mandatory components of the confirmatory study design. Additionally, the anatomical discrepancy between sampling sites, maxillary sinus mucosa in ORS patients versus nasal septal mucosa in controls, represents a methodological limitation, as these tissues differ in their microenvironmental context. Future confirmatory studies should adopt anatomically matched sampling strategies, such as sinus lavage fluid collection or middle meatal cytobrush sampling in control subjects, to ensure biological comparability between groups.

## 5. Conclusions

This pilot, proof-of-concept study achieved its two primary objectives: establishing that genotyping-grade genomic DNA can be recovered and *TAS2R38* genotyped from acutely inflamed maxillary sinus mucosa, and generating preliminary non-taster prevalence estimates to inform sample size planning for a confirmatory trial (with HWE confirmed as a routine quality control step). The primary analysis showed no significant association between AVI/AVI non-taster status and ORS susceptibility, a finding unchanged in the sensitivity analysis retaining unclassifiable genotypes. The absence of a statistically significant association between the *TAS2R38* genotype and ORS susceptibility is the expected outcome of an exploratory, underpowered investigation and is uninformative with respect to the underlying biological hypothesis in either direction. The demonstrated feasibility and the established biological rationale together support the evaluation of this question in a prospective, adequately powered multicenter study, laying the foundation for future investigation of taste receptor genetics in ORS pathogenesis and potentially personalized therapeutic strategies.

## Figures and Tables

**Table 1:** Sample size estimates for a confirmatory study, by assumed odds ratio and power level.

Assumed OR	Non-Taster Prevalence in Controls	Power 80% (per Group)	Power 90% (per Group)	Attrition-Adjusted 80% (per Group)	Attrition-Adjusted 90% (per Group)
1.36 (observed)	25.0%	857	1136	1072 (2144 total)	1420 (2840 total)
2.0 (literature-based, primary)	25.0%	165	216	207 (414 total)	270 (540 total)
2.5 (literature-based, upper)	25.0%	93	122	117 (234 total)	153 (306 total)
3.0	25.0%	64	85	80 (160 total)	107 (214 total)

Legend: All estimates are based on a two-sided Fisher’s exact test at α = 0.05, assuming a baseline non-taster (AVI/AVI) prevalence of 25.0% in controls. The “Power 80%/90% (per group)” columns give the analyzable sample size—participants with fully classifiable canonical TAS2R38 genotypes; the “Attrition-adjusted” columns give the corresponding recruitment target after a 20% correction for unclassifiable (mixed and incomplete) genotypes. Totals (both groups combined) are shown in parentheses. The OR = 1.36 row reflects the effect size observed in the present pilot and is shown for reference only; OR = 2.0 is the primary planning assumption.

## Data Availability

The original contributions presented in this study are included in the article. Further inquiries can be directed to the corresponding author.
